# Dietary Supplementation of Two-Stage Fermented Feather-Soybean Meal Product on Growth Performance and Immunity in Finishing Pigs

**DOI:** 10.3390/ani11061527

**Published:** 2021-05-24

**Authors:** Hsien-Juang Huang, Bor-Chun Weng, Yan-Der Hsuuw, Yueh-Sheng Lee, Kuo-Lung Chen

**Affiliations:** 1 Kaohsiung Animal Propagation Station, COA-LRI, Pingtung 912013, Taiwan; hjhuang@mail.tlri.gov.tw; 2 The Graduate Institute of Biotechnology, National Pingtung University of Science and Technology, Pingtung 912301, Taiwan; hsuuw@mail.npust.edu.tw; 3 Department of Microbiology, Immunology and Biopharmaceuticals, National Chiayi University, Chiayi 600355, Taiwan; brian@mail.ncyu.edu.tw; 4 The Graduate Institute of Animal Sciences, National Chiayi University, Chiayi 600355, Taiwan; lopqkojhog@gmail.com

**Keywords:** finishing pig, growth performance, immunity, blood biochemicals, feather meal, fermentation, probiotics

## Abstract

**Simple Summary:**

Feathers contain around 90% of keratin which compose of unbalanced amino acids with low digestibility and limiting the usage in monogastric animal diets. To improve the nutrient value of feather through fermentation using keratinase-producing microbes pose a high economic potential. This study investigates the effects of two-stage fermented feather-soybean meal product (TSFP) on growth performance, blood characteristics, and immunity of finishing pigs. In growth performance, 2.5–5% TSFP promotes the average daily feed intake and feed conversion rate with a best performance of 5%. In blood characteristics, 5% TSFP increased HDL-C, and decreased LDL-C and blood urea nitrogen content. In immunity, 5% TSFP increased lymphoblastogenesis stimulated by lipopolysaccharide and concanavalin A, and promoting IFN-γ, IgA productions, and phagocytic cells oxygen burst capacity. It appears that TSFP improves the growth performance and immunity of finishing pigs.

**Abstract:**

This study investigates the effects of two-stage fermented feather meal-soybean meal product (TSFP) on growth performance, blood characteristics, and immunity of finishing pigs. Firstly, feather meal-soybean meal is subjected to aerobic fermentation with *Bacillus subtilis* var. natto N21, *B. subtilis* Da2 and Da15, *B. amyloliquefaciens* Da6, Da16 for two days, and anaerobic fermentation with *B. coagulans* L12 for three days. Then, the fermented product is air-dried into an end product—TSFP. Eighty hybrid pigs (Duroc x KHAPS) with equal numbers of both sexes are randomly assigned into 3% fish meal, 0%, 2.5%, or 5.0% TSFP groups with five replicates per group. Our results show that the average daily feed intake and feed conversion rate of TSFP groups are significantly better than the other groups at 0–3 weeks (*p* < 0.05). The 5% TSFP group significantly increased HDL-C in the blood (*p* < 0.05), and decreased LDL-C and blood urea nitrogen content (*p* < 0.05). The lipopolysaccharide (LPS) and concanavalin A (ConA) in 5% TSFP group and interferon-γ (IFN-γ) content in 2.5% and 5% TSFP groups are significantly higher than the other groups (*p* < 0.05). The phagocytic oxygen burst capacity and serum IgA content of the 5% TSFP group are significantly higher than those of the fishmeal group (*p* < 0.05). The CD3, CD4, and CD4 + CD8 + T cells subsets in 2.5% and 5% TSFP groups are significantly higher than the control group (*p* < 0.05). In conclusion, TSFP has a positive effect on the growth performance and immunity of finishing pigs with the best performance on 5% TSFP.

## 1. Introduction

Feathers contain up to 85% of crude protein (CP) with primarily (around 90%) keratin which enriched disulfide bond and hydrophobic properties with unbalanced amino acid composition impacted on digestibility [1,2,3,4,5,6]; therefore, limiting usage of feathers in monogastric animal diets. Pressurized cooking processing to hydrolyzed feather meal is the primary processing method currently. The process has often resulted in denature and loss of available amino acids and further impact its nutritional value for monogastric animals [5,6]. It, therefore, has been recommended to limit the use of feather meal to a maximum of 5% in growing pig diets [7].

Process feather meal using microbial fermentation not only promotes the nutritional value of matrix by decreasing anti-nutritional factors and degrading complex proteins and carbohydrates [8,9,10], but also improves feed palatability and preservation period; hence, it has been widely adopted in animal feed for many years [11,12,13]. Moreover, Nudda et al. (2019) have reported that to use of agriculture by-products in animal health promotion, reduction of agri-waste are important strategies in sustainable agriculture [14]. However, fermentation in feather meal for pig feeds is relatively rare and not available in the literature. Some of our previous studies reported that using keratinase-producing microbes can improve the value of feather meal to be similar to soybean meal [15,16,17].

Our previous broilers study has reported that the complete poultry feed ration under the first stage 2-days aerobic fermentation with *Bacillus subtilis* var. natto N21 (N21) and second stage 3-days anaerobic fermentation with *Bacillus coagulans* L12 (L12) fed chicken had significantly improved growth performance as compared with the control group on the unfermented feed [18]. In a pig study, Huang et al. (2014) mixed feather meal and soybean meal at a ratio of 2:3 to as fermentation substrate, and using the same method as Yeh et al. (2018) to produce a two-stage fermented product [18,19]. Adding 5% two-stage fermented product in growing pigs feed, which affects promoting growth performance, and completely replaces high-quality fish meal [19]. 

N21 is mainly used to decompose plant protein. In addition to the N21 originally selected for plant protein sources, the selected keratinase producing *Bacillus subtilis* strains Da2 (Da2), Da15 (Da15), and *Bacillus amyloliquefaciens* strains Da6 (Da6), Da16 (Da16) were serially composed together into a bacterial mixture of five-*Bacillus* strains for a first stage production. The purpose of this study was to examine the effects of feather under hydrolysis and keratinase activity of the *Bacillus*-mixture fermentation in the first stage production. Secondly, to investigate the effects of two-stage mixed fermented products of feather-soybean meal on growth performance, blood characteristics, and immunity of finishing pigs.

## 2. Materials and Methods

### 2.1. Feathers Degradability

Adding 50 mL TSB medium (Tryptone Soya Broth, HIMEDIA^®^, Shenzhen, China) and 3% feathers into a 250 mL flat-bottomed Erlenmeyer flask. After sterilization (121 °C, 20 min), inoculate 0%, 5% *Bacillus subtilis* var. natto N21 (N21) and mixed strains culture (N21, *B. subtilis* Da2 (Da2) and Da15 (Da15), *B. amyloliquefaciens* Da6 (Da6), Da16 (Da16)) (10^9^ cfu/mL), and incubate at 37 °C, 100 rpm, for 0, 24, 48 and 72 h. The hydrolysate was passed through a filter paper (No. 1 filter paper, ADVANTEC^®^, Tokyo, Japan) to remove unhydrolyzed feathers at different times. The feather degradability was calculated using the following formula:

Feather degradation rate (%) = (A − B − C)/A × 100(1)

where A is the dry weight of the feathers before degradation, B is the dry weight of the feathers and filter after degradation, and C is the dry weight of the filter.

### 2.2. Keratinolytic Activity Assay

The extracted culture broth was filtered with a 0.2 μm Analytical filter funnel (NALGENE^®^, Shanghai, China). Mixed 700 μL of the filtrate with 6.3 mL of 10 mM acetate buffer (pH 5.5) and used ultra-concentrated centrifuge tube (Amicon Ultra-15 Centrifugal Filter Units, 10 kDa, Millipore) to concentrate at 3500× *g* for 30 min. The filtrate was concentrated to a volume of 250 μL, and diluted 4-fold into an enzyme solution (ES). Mix 70 μL of the substrate solution (2.1 mg Azokeratin + 14 μL 0.5 M phosphate buffer + 56 μL of 2D water) with 40 μL of ES. The reaction was incubated at 50 °C for 60 min. The reaction was terminated by adding 25 μL of 4 M NaOH and was centrifuged at 8000× *g* for 20 min; then, 100 μL of the supernatant was added to the wells of the ELISA plate sequentially, and the absorbance at 450 nm was measured. Using 200 μL of 1N NaOH solution as blank, and the enzyme activity was calculated using the following Formula (2):

Keratinolytic activity (U/mL) = (OD − Blank)/(Reaction time × 0.001) × concentrated fold(2)

### 2.3. Two-Stage Fermented Product (TSFP) Preparation

The TSFP preparation and analysis followed the description of Yeh et al. [18] with a minor modification. Da2, Da6, Da15, Da16, and N21, having strong protein decomposition ability, were used as the first-stage fermentation strains, and then *B. coagulans* L12 (L12) with strong acid-producing ability was added as the second-stage fermentation strain. Mixed soybean meal and feather meal at a ratio of 1:1 was supplied as fermentation substrate. The substrate was sterilized at 121 °C for 30 min, and cooled it down to 45 °C. Each of five *Bacillus* strains at 10^6^ CFU/g of substrate was premixed and inoculated together with 50% *w/w* sterilized water to ferment aerobically at 37 °C for 2 days at the first stage; subsequently, the L12 at 10^6^ CFU/g of the substrate was added to ferment anaerobically at 28 °C for additional 5 days at the second stage. The fermented product was dried using an oven. The moisture content of the final product (TSFP) was below 12%, and 3 batches were produced for the current study.

### 2.4. Animal Management and Experimental Design

All the procedures used in this experiment were approved by the Institutional Animal Care and Use Committee of Kaohsiung Animal Propagation Station (IACUC, protocol number 101005). Eighty hybrid pigs (Duroc x KHAPS) [20] with equal numbers of both sexes were randomly assigned into 3% fish meal, 0% (as control), 2.5% or 5.0% TSFP groups. Each treatment had 5 replicates. The experimental period was 9 weeks. The hybrid pigs were raised in floor pens (3.84 m × 2.56 m) during the entire experiment. Proximate feed analysis and amino acid analyses followed the description of Yeh et al. [18] to analyze the moisture, crude protein, calcium, phosphorus, lysine, and methionine of feed. Feed composition is shown in Table 1. Feed and water were provided *ad libitum* throughout the experimental period. Bodyweight and feed intake were recorded and monitored the weight gain and feed conversion weekly throughout the experiment.

### 2.5. Hematology and Blood Biochemistry

At the end of the experiment, blood samples were obtained from the jugular vein with an EDTA vacutainer (BD Vacutainer^TM^, Avenue Broken Bow, NE, USA). The blood samples were stored at 4 °C until ready for analysis. The hematology, including white blood cells (WBC), neutrophils (NE), lymphocytes (LY), monocytes (MO), eosinophils (EO), basophils (BA), red blood cells (RBC), hemoglobin (Hb), hematocrit (Hct), mean corpuscle volume (MCV), mean red blood cell hemoglobin (MCH), mean red blood cell hemoglobin concentration (MCHC), red cell distribution width (RDW), platelet count (PLT), and mean platelet volume (MPV), was determined using an automatic hematological analyzer (HEMAVET, USA). Plasma was obtained by centrifugation at 2500× *g* for 30 min at 4 °C and stored at −20 °C for later determination of the blood biochemistry. The blood biochemistry of the plasma, including total protein (TP), cholesterol (CHOL), high-density lipoprotein-cholesterol (HDL-C), low-density lipoprotein-cholesterol (LDL-C), glucose (GLU), aspartate aminotransferase (AST), alanine aminotransferase (ALT), alkaline phosphatase (ALP) and blood urea nitrogen (BUN), was determined using a Blood Analyzer (Express Plus, Bayer, MA, USA). The total cholesterol (CHOL), high-density lipoprotein-cholesterol (HDL-C), and low-density lipoprotein-cholesterol (LDL-C) determinations used the method of Rifai et al. [21]. The serum glucose was estimated by the oxidase method reported by a previous study [22]. The serum urea was assayed by the method of Tomas et al. [23], and serum AST and ALT were assayed using the method of Moss and Henderson [24].

### 2.6. Blood Immunoglobulin Level

Total porcine immunoglobulin was determined in the serum as previously described by Mizumachi et al. [25]. The jugular blood was collected in an EDTA vacutainer tube and then centrifuged at 10,000× *g* for 2 min after clotting and stored individually at −80 °C. The serum antibodies containing immunoglobulin M (IgM), immunoglobulin G (IgG), and immunoglobulin A (IgA) were measured by a sandwich enzyme-linked immunosorbent assay (ELISA). Briefly, microtiter plate wells (Maxisorp; Nunc, Roskilde, Denmark) were coated overnight at 4 °C with goat anti-porcine IgM, IgG, or IgA (Bethyl Laboratories, Montgomery, TX, USA). The coated wells were washed with phosphate-buffered saline (PBS) containing 0.05% Tween 20 (PBST) and blocked with 1% bovine serum albumin (BSA) in PBS for 30 min. After washing with PBST, the properly diluted samples were added and incubated for 2 h at room temperature (RT). Subsequently, the wells were treated with horseradish peroxidase-conjugated goat anti-porcine IgM, IgG, or IgA (Bethyl Laboratories, USA) for 1 h at RT. The wells were washed, and a 3,3′,5,5′-tetramethylbenzidine (TMB) solution (KPL, Gaithersburg, MD, USA) was added to each well as a substrate. After 30 min of incubation at RT, the reaction was stopped by adding 1 M dihydrogen phosphate (H_2_PO_3_). The absorbance was measured at 450 nm using a microplate reader (Original multi scan, Thermo, Waltham, MA, USA).

### 2.7. Cytokine Production

Determination of cytokine production in whole blood was described by Edfors-Lilja et al. [26]. Heparinized whole blood was diluted to 1:50 for the detection of interferon-γ (IFN-γ) by the culture medium (Roswell Park Memorial Institute-1640; RPMI-1640) containing 50 μM 2-mercaptoethanol, 10 Mm 4-(2-hydroxyethyl)-1-piperazineethanesulfonic acid (HEPES), 10 U/mL penicillin and 100 μg/mL streptomycin. The diluted whole blood samples were seeded into 24-well plates and cultured in a humid incubator. The incubator was maintained at 37 °C and 5% CO_2_ gas. The culture supernatant was collected after 72 h for determination of IFN-γ. The cytokine levels were determined by commercial ELISA reagents (R&D Systems, Minneapolis, MN, USA and IFN-γ; PharMingen, San Diego, CA, USA) according to the manufacturer’s procedures. Color changes were detected at OD = 450 nm and 550 nm with a microplate reader (Multiskan Ex Microplate Reader, Thermo, Waltham, MA, USA).

### 2.8. Isolation of Peripheral Blood Mononuclear Cells and Granulocytes

The jugular blood collected in the EDTA tube was centrifuged at 400× *g* for 10 min at 4 °C, and the plasma was then removed for the later assays. The cell portion was diluted with RPMI-1640 (1:2) and layered onto Ficoll (Histopaque-1077, Sigma-Aldrich, St. Louis, MO, USA) for the density gradient separation. After 30 min, the samples were centrifuged at 450 × g at room temperature, and the peripheral blood mononuclear cells (PBMCs) were collected. After a wash with cold PBS, the cells were subjected to a live/dead count by a trypan blue exclusion method with a hemocytometer under a microscope as described elsewhere. After removal of the PBMCs from the samples, the red blood cell portions were subsequently lysed by commercial RBC-lysis buffer (BioLegend, San Diego, CA, USA) to remove red blood cells and harvest granulocytes with centrifugation. Purified granulocytes were then counted as described and subjected to a later assay.

### 2.9. Phagocytosis of Granulocyte

DioC18 (0.25 mg/mL; Invitrogen, Waltham, MA, USA) labeled *Escherichia coli* (ATCC 25922) was suspended in 0.5 mL of Hank’s balanced salt solution (HBSS) and used for the analysis of phagocytosis. Granulocytes of 1 × 106 each were preseeded in a 96-well plate and then cocultured with fluorescently labeled bacteria at 1 × 107 DioC18-labeled *E. coli.* in a PBS solution at 37 °C for 90 min. By the end of the coincubation, 100 μL of trypan blue (1.25 mg/mL) were added to quench the residual DioC18-labeled *E. coli.* Phagocytosis of the granulocytes was determined by flow cytometry (Becton Dickinson FACSCalibur^TM^, Franklin Lakes, NJ, USA).

### 2.10. Oxidative Burst Measurement

At a similar setting as described in the phagocytosis, the granulocytes were coincubated with unlabeled *E. coli.* in a 37 °C incubator for 90 min, and the intracellular ROS was determined by adding 2′,7′-dichlorofluorescin-diacetate (DCF-DA) as described by Ciapetti et al. [27]. The generated DCF was directly proportional to the reactive oxygen species (ROS) as the process of the oxidative burst of the granulocytes was measured by flow cytometer.

### 2.11. Assessment of Lymphoblastogenesis

As described by Weng et al. [28], briefly, isolated live PBMCs were diluted in 1 × 10^6^/mL and seeded onto a 96-well plate. Specific mitogens, all purchased from Sigma, USA, including 25 μg/mL concanavalin A, 20 μg/mL lipopolysaccharide, or 50 ng/mL phorbol 12-myristate 13-acetate plus 250 ng/mL ionomycin, were added to stimulate specific lymphocyte proliferation. Alamar Blue (Serotec Co., Oxford, UK) was added in the last 24 h of an entire 72 h culture at 37 °C in a 5% CO_2_ humidified incubator. The changes of specific fluorescence were measured by a microplate reader (FLX800, Bio-Tek Instruments, Inc., Winooski, VT, USA).

### 2.12. Lymphocyte Subpopulation Analysis

Fluorescence-labeled primary antibodies were used for the swine lymphocyte subpopulation, including the total T cells, T helper cells, and cytotoxic T cells, and the CD4CD8 double positive population by a flow cytometry. All the fluorescence-labeled monoclonal antibodies were purchased from Serotec Company (Oxford, UK). Briefly, each of 1 × 10^6^ PBMCs samples was incubated with 10 μL of specific fluorescence-conjugated monoclonal antibodies in refrigeration avoiding light for 90 min, and procedures were followed as recommended by the manufacturer. The lymphocyte subpopulations were then determined by flow cytometry and analyzed by CellQuest software (Becton Dickinson FACS Calibur^TM^, CA, USA).

### 2.13. Statistical Analysis

For analyses of feather degradation and keratinase activity, each test had 3 replicates. For analysis of growth performance, a single pen (*n =* 5) was considered as the experimental unit. For analyses of hematology, blood biochemistry, and immune characteristics of blood, individual pigs (*n =* 20) were considered as experimental units.

The data were calculated using the General Linear Model (GLM) procedure [29], and the groups were compared using a one-way ANOVA with a Tukey post hoc test, where *p* < 0.05 indicated a statistically significant difference.

## 3. Results

### 3.1. Feather Degradation and Keratinase Activity by the Bacillus-Mixture Fermentation at Stage One

Table 2 present the results of fermentation on feather hydrolysis and keratinase activity. Fermentation with N21 alone had significantly (*p* < 0.05) increased feather degradation rate and keratinase activity, while the fermentation of mixed *Bacillus* strains group resulted in the highest (*p* < 0.05) feather degradation rate and keratinase activity over a 72-h period time.

### 3.2. The Physiochemical Characterizations and Nutrient Composition of TSFP

Table 3 shows the results of the TSFP physiochemical analysis. The pH value and bacterial counts showed a significant increase in the first stage fermentation, then reduced to 5.83 in the pH value, and increased to 8.52 log CFU/g in the total *Lactobacillus* number, respectively, during the subsequently 3-days secondary anaerobic fermentation. Then the *Bacillus*-like bacteria further reduced to 7.52 log CFU/g after dehydration (dry powder), while the total lactic acid bacteria remained high counts at 7.32 log CFU/g. The nutrients composition did not show any effect by fermentation.

### 3.3. Growth Performance

Table 4 presents the effect of dietary TSFP level on the growth performance of finishing pig. At weeks 0 to 3, the ADG and FCR of 2.5% group were comparatively to 5% group and significantly (*p* < 0.05) better than the other groups, while only the 5% supplemented group showed superior (*p* < 0.05) results with extending growth periods of 3–6 weeks and 6–9 weeks. Pig diet supplemented with TSFP showed an overall increasing bodyweight gain, feed intake, and FCR than the 3% fish meal diet and basal diet groups for the entire nine weeks feeding trial. Nevertheless, the 5% TSFP supplemented group showed better (*p* < 0.05) growth performance than the 2.5% group.

### 3.4. Hematology

Table 5 showed the effect of dietary supplementation of TSFP on hematology. The amounts of WBC, NE, EO, MO, BA, LY, RBC, Hb, Hct, MCV, MCH, RDW, PLT, and MPV did not significantly (*p* > 0.05) different among groups. The results indicated no detrimental effects found in the dietary intervention.

### 3.5. Blood Biochemistry

Table 6 showed the effect of TSFP supplementation on blood biochemistry. Dietary supplementation of 5% TSFP had elevated HDL-C and reduced LDL-C, and BUN levels, as compared with 3% Fish meal and control groups (*p* < 0.05). 

### 3.6. Immune Characteristics

Table 7 showed that TSFP supplementation affected immunity. Pigs supplemented with 5% TSFP had significantly (*p* < 0.05) activated higher lymphoblastogenesis by either LPS or ConA, and the IFN-γ production was the highest (*p* < 0.05) than other groups. Moreover, the IgA production and the oxidative burst were higher (*p* < 0.05) in the 5% group than those of the fish meal group. The analysis of T cells subset populations revealed the ratios of CD3, CD4, CD4^+^ CD8^+^ T cells of the 2.5% and 5% groups were significantly (*p* < 0.05) higher than the control group. 

## 4. Discussion

Notably, the Mix fermentation group had a 31% increase in the keratinase activity at the first 24 h of fermentation. Peng et al. (2019) has reported that coincubation with *B. licheniformis* BBE11-1 and *S. maltophilia* BBE11-1 improved feather degradation [30]. Similarly, the feather degradation has increased 74% by 48 h in current settings, which fermented aerobically by the mixture of five selected Bacillus strains at the first stage.

*Bacillus* spp. tends to grow in pH neutral environment [31]. Along with the proliferation of the bacteria, the increasing alkaline metabolites raise the environmental pH value [32]. This agrees with current results that the pH values rose from 5.76 to 7.60 during the first stage fermentation. At the second stage of fermentation, the anaerobic fermentation with L12 has acidified the fermented product to pH = 5.83. Fermented products then dried in an oven, which did not further influence the pH value. Yeh et al. (2018) has indicated that the lactic acids were primarily produced at the second stage fermentation. Since the organic acids were not volatile, the pH remained the same between the liquid fermented product and the dried end product [18]. Moreover, the *Bacillus* spp. and *Bacillus coagulans* can form spores [33,34], which can resist high pressure and low pH [35]. L12 is a lactic acid-producing bacteria and is identified as *B. coagulans*. Although the *Bacillus* spp. had less impacted by low temperature (55 °C) drying, the overall lactic acid bacteria count had reduced to 7.52 log CFU/g.

Compared to 3% fish meal on the growth performance, the different levels of TFSP have demonstrated similar or even better effects in the current study. It has been reported that under the same levels of dietary crude protein and energy with different protein sources; soybean meal vs. fish meal has exhibited similar effects on average daily weight gain and feed efficiency in fishing pigs [36]. The growth performance did not show significantly different between the fish meal group and control group in the current study, suggesting the fish meal supplementation might not be essentially required in finishing pig. Our previous broilers study, Yeh et al. (2018) also showed dietary supplementation of a 5% two-stage fermented product has improved growth performance and ileal amino acid digestibility [18]. In general, the fermented product derived from plant protein has exhibited beneficial effects on growth performance and immunity in pig studies [36,37]. However, the study of utilization fermented feather meal is rare. Our previous trial has demonstrated that dietary supplementation of a 5% two-stage fermented feather-soybean meal performs comparably as a high-quality fish meal supplementation in the growth performance of growing pigs [19]. In this study, the fermented product was obtained by a two-stage fermentation of the first stage aerobic incubation using a mixture of *Bacillus* spp. with the substrate of feather meal and soybean meal, and the subsequent second stage anaerobic fermentation with L12. Supplementation of 2.5% TSFP in pig diet had improved ADG and FCR compared to fish meal supplementation. In addition, the 5% fermentation powder supplied group showed 18.4% and 18.7% increases in ADG and FCR, respectively. On-farm observations also found a better appetite and feces structure without the diarrhea incidence. Notable healthy appearance and improved wellbeing were attained. The current TSFP may bring beneficial for pig farming.

All hematological values were within normal ranges [38]. Hung et al. (2006) has also shown that dietary supplementation of 3% fermented soybean meal in fishing pigs did not affect hematological parameters [39]. Similar results were obtained in the current study in an indication of no adverse implication. Probiotics can effectively improve blood lipidemia by reducing blood total cholesterol, LDL, and triglycerides levels, while increasing liver-derived HDL-C [40]. Current TSFP content-rich *Bacillus* spp. with lactic acid-producing bacteria. When pigs were supplemented with 5%, TSFP did not affect blood total cholesterol. The lower LDL-C and higher HDL-C levels were observed in the 5% TSFP group. HDL-C synthesized in the liver and intestine is responsible for transport cholesterol back to liver for metabolism, while LDL-C is to carry cholesterol for cells and tissue utilization. The LDL-C is known notoriously related to atherosclerosis and other vascular diseases, such as thrombosis. Feed supplemented with the TSFP may exhibit beneficial effects on cholesterol forming, transportation, and metabolism in pigs.

BUN is metabolites of proteins and is excreted by the kidney through urine, which reflects the function of the kidney. Several factors may lead to increase BUN levels, especially protein mal-metabolism. In the current study, elevating BUN level might be associated with enhanced protein digestion and absorption. Two-stages fermentation may degrade proteins into small peptides, or amino acids leads to better protein utilization and absorption [18,41]. In the current study, the 5% TSFP group increased ADFI and improved the FCR (Table 4). All the results demonstrated that TSFP has positively affect digestion, metabolism, and physiological parameters. Clinical analysis on ALP, ALT, and AST levels did not show significant differences among groups, and all fall within normal ranges. It appears that dietary supplementation of TSFP had no adverse effect.

Mitogenic stimulations by LPS or ConA elicited non-specific B cell and T cell clones proliferations, respectively. Lymphocyte specificity is clonally distributed—upon encounter antigens, the higher lymphocyte proliferating activity implies better adaptive immune response. The cytokine, IFN-γ is essential in defending against viral infection, which is important in cell-mediated immunity. The 5% TSFP group had shown to boost cell-mediated immunity, which significantly (*p* < 0.05) increased the ratio of CD4 T cells and production of IFN-γ. Consistent with the findings of enhanced cell-mediated immunity, the innate immune functions of phagocytosis activity and the oxidative burst of phagocytic cells were both upregulated in the 5% group. 

The phagocytic coordination serves as the first defense line during bacterial infection, and the ROS generated during oxidative bursts is responsible for killing phagocytosed bacteria [42]. Cha et al., (2013) reported that dietary supplement with 0.5% *Bacillus *spp. has increased oxidative burst, improved immunity, and feed efficiency, complying with current findings [43]. Moreover, the IgA production of the 5% TSFP group was significantly (*p* < 0.05) higher than that of the 3% fish meal supplemented group. Steiner (2006) states that oral supplements with probiotics can increase immunoglobulins production [44]. Furthermore, Lee et al. (2014) show piglet diet supplied with 0.45% *Bacillus subtilis* has increase IgA and IgG productions [45]. The current results demonstrated supplementation of TSFP in pigs may boost humoral immunity by increasing immunoglobulin production.

## 5. Conclusions

In conclusion, cocultivating feathers with mixed *Bacillus* strains show an optimum feather degradation rate and keratinase activity. Dietary 2.5–5% TSFP promotes growth performance and boosts immunity in finishing pigs. Whereas dietary inclusion of the 5% TSFP has obtained the best overall performances.

## Figures and Tables

**Table 1:** Composition of experiment diets (as-fed basis).

Ingredient, %	3% Fish Meal	TSFP, %		
		0	2.5	5
Corn meal	76.44	73.84	74.95	75.71
Soybean meal, 44%	17.96	22.84	18.98	15.50
Fish meal (Peru), 65%	3.00	0.00	0.00	0.00
TSFP, 62%	0.00	0.00	2.50	5.00
Ground limestone	0.85	0.90	0.90	0.90
Dicalcium phosphate	0.64	1.00	1.04	1.05
Soybean oil	0.56	0.82	0.98	1.14
Salt	0.25	0.25	0.25	0.25
Choline chloride 50%	0.10	0.10	0.10	0.10
L-Lys·HCl (78%)	0.00	0.02	0.07	0.11
DL-Met	0.00	0.03	0.03	0.04
Vitamin premix ^1^	0.10	0.10	0.10	0.10
Micromineral premix ^2^	0.10	0.10	0.10	0.10
Total	100.0	100.0	100.0	100.0
Analysis				
CP, %	15.39	15.56	15.57	15.52
Ca, %	0.63	0.66	0.67	0.67
Total P, %	0.49	0.51	0.51	0.52
Lys, %	0.81	0.85	0.85	0.84
Met, %	0.30	0.31	0.32	0.32

^1^ Vitamin supplied the following per kilogram of premix: Vitamin A, 5000 IU; vitamin D_3_, 1500 IU; vitamin E, 40 mg; vitamin K, 3 mg; vitamin B_1_, 2.6 mg; vitamin B_12_, 0.04 mg; niacin, 35 mg; pantothenic acid, 23 mg. ^2^ Mineral supplied the following per kilogram of premix: Fe (FeSO_4_·7H_2_O, 20.09% of Fe), 217 mg; Cu (CuSO_4_·5H_2_O, 25.45% of Cu), 125 mg; Mn (MnSO_4_·H_2_O, 32.49% of Mn), 40 mg; Zn (ZnSO_4_, 80.35% of Zn), 110 mg; Se (NaSeO_3_, 45.56% of Se), 0.36 mg; Co (CoSO_4_·H_2_O, 32% of Co), 0.7 mg.

**Table 2:** The effect of liquid fermentation on feather degradation rate and keratinase activity.

Time	Control	N21	Mix	SEM	*p*-Value
Feather degradation rate, %					
24 h	2.85 ^c^	30.6 ^b^	61.6 ^a^	0.35	<0.0001
48 h	3.35 ^c^	60.2 ^b^	74.2 ^a^	0.42	<0.0001
72 h	4.33 ^c^	68.1 ^b^	79.1 ^a^	0.36	<0.0001
Keratinase activity, U/mL					
24 h	22.4 ^c^	414 ^b^	634 ^a^	4.54	<0.0001
48 h	17.9 ^c^	409 ^b^	555 ^a^	6.26	<0.0001
72 h	23.6 ^c^	401 ^b^	539 ^a^	3.03	<0.0001

*n =* 3. ^a,b,c^ Means in the same row with different superscripts are significantly different (*p* < 0.05).

**Table 3:** Physical and chemical analysis of TSFP.

Items	Means ± SE
pH value	
Initial condition	5.76 ± 0.01
First stage fermentation	7.60 ± 0.01
Second stage fermentation	5.83 ± 0.01
Dry powder	5.97 ± 0.04
*Bacillus*-like, log CFU/g feed	
Initial condition	1.02 ± 0.04
First stage fermentation	8.54 ±0.07
Second stage fermentation	7.53 ±0.06
Dry powder	7.52 ± 0.01
Total lactic acid bacteria, log CFU/g Feed	
Second stage fermentation	8.52 ± 0.01
Dry powder	7.32 ± 0.01
Nutrient composition of dry powder	
Moisture, %	5.4 ± 0.05
Crude ash,%/DM	4.63 ± 0.11
Crude protein, %/DM	62.75 ± 0.92
Gross energy, kcal/kg/DM	3156 ± 148
Calcium, Ca %/DM	0.25 ± 0.01
Total Phosphate, TP %/DM	0.54 ± 0.01

**Table 4:** Effects of TSFP on growth performance of finishing pigs.

Period, WK.	3% Fish Meal	TSFP, %			SEM
		0	2.5	5	
Bodyweight (BW), kg					
0	78.17	78.44	78.13	78.15	0.65
3	88.42 ^b^	89.03 ^b^	91.24 ^a^	91.22 ^a^	0.76
6	100.03 ^c^	101.72 ^c^	104.56 ^b^	106.58 ^a^	1.01
9	111.24 ^c^	111.90 ^c^	115.58 ^b^	118.79 ^a^	1.20
Average daily gain (ADG), kg					
0–3	0.49 ^b^	0.50 ^b^	0.62 ^a^	0.62 ^a^	0.04
3–6	0.55 ^b^	0.60 ^b^	0.63 ^a,b^	0.73 ^a^	0.05
6–9	0.54 ^a,b^	0.48 ^b^	0.52 ^a,b^	0.58 ^a^	0.04
0–9	0.52 ^c^	0.53 ^c^	0.59 ^b^	0.65 ^a^	0.02
Average daily feed intake (ADFI), kg					
0–3	1.83 ^b^	1.85 ^b^	1.90 ^a^	1.84 ^b^	0.03
3–6	1.84 ^c^	1.88 ^b^	1.90 ^b^	1.95 ^a^	0.02
6–9	1.91 ^b^	1.90 ^b^	1.92 ^b^	1.96 ^a^	0.02
0–9	1.86 ^b^	1.87 ^b^	1.91 ^a^	1.92 ^a^	0.01
Feed conversion rate (FCR), ADFI/ADG					
0–3	3.85 ^a^	3.73 ^a^	3.10 ^b^	3.04 ^b^	0.26
3–6	3.46 ^a^	3.13 ^a,b^	3.08 ^a,b^	2.74 ^b^	0.25
6–9	3.65 ^a,b^	4.01 ^a^	3.72 ^a,b^	3.54 ^b^	0.23
0–9	3.58 ^c^	3.54 ^c^	3.23 ^b^	2.98 ^a^	0.12

*n =* 5. ^a,b,c^ Means with the same letter in the row are not significantly different (*p* < 0.05).

**Table 5:** Effect of TSFP on hematological traits of finishing pigs.

Items	3% Fish Meal	TSFP, %			SEM
		0	2.5	5	
WBC, 10^3^/μL	17.61	20.16	19.63	19.29	2.06
NE, % × 10^3^/μL	8.4	9.42	7.71	7.86	1.20
LY, % × 10^3^/mL	7.38	8.13	9.33	9.38	0.98
MO, % × 10^3^/μL	0.82	1.12	1.04	1.07	0.15
EO, % × 10^3^/μL	0.93	0.96	1.28	1.02	0.25
BA, % × 10^3^/μL	0.11	0.11	0.11	0.11	0.02
NE, %	45.18	47.74	39.4	40.9	4.05
LY, %	44.66	41.85	47.45	48.93	3.85
MO, %	4.54	5.44	5.25	5.65	0.70
EO, %	5.49	4.43	7.2	6.5	2.20
BA, %	0.37	0.55	0.52	0.43	0.09
RBC, 10^6^/mL	6.87	6.08	7.1	6.97	0.70
Hb, g/dL	10.56	10.31	10.39	10.5	0.78
HCT, %	38.13	43.66	41.38	47.78	4.32
MCV, Fl	63.42	62.01	65.29	67.02	3.79
MCH, pG	14.62	13.8	14.28	14.29	0.87
MCHC, g/dL	22.36	20.63	20.69	19.84	1.03
RDW, %	22.05	22.09	21.95	21.53	0.66
PLT, 10^3^/mL	384.5	349	329.5	348	63.26
MPV, fL	6.44	5.45	5.53	5.78	1.15

*n =* 20. WBC, white blood cells; NE, neutrophils; LY, lymphocyte; MO, monocyte; EO, eosinophil; BA, basophilic ball; RBC, red blood cell; Hb, hemoglobin; Hct, hematocrit; MCV(fL), mean corpuscle volume; MCH(pg), mean red blood cell hemoglobin; MCHC(g/dL), mean red blood cell hemoglobin concentration; RDW, red cell distribution width; PLT, platelet count; MPV, mean platelet volume.

**Table 6:** Effect of TSFP on blood biochemistry of finishing pigs.

Items	3% Fish Meal	TSFP, %			SEM
		0	2.5	5	
TP, g/dL	9.83	10.84	11.21	11.29	0.77
CHOL, mg/dL	120.85	119.04	118.79	113.11	6.98
HDL-C, mg/dL	58.19 ^b^	59.08 ^b^	63.94 ^a,b^	67.90 ^a^	2.98
LDL-C, mg/dL	42.63 ^a^	37.36 ^a^	40.40 ^a^	30.14 ^b^	3.99
GLU, mg/dL	46.38	47.13	46.9	47.9	9.72
BUN (U/L)	17.95 ^a^	16.00 ^a^	15.03 ^a^	11.87 ^b^	2.16
AST (U/L)	12.73	14.07	15.39	14.76	3.42
ALT (U/L)	26.81	23.65	24.73	21.01	3.46
ALP (U/L)	34.39	33.55	33.02	31.36	3.65

*n =* 20. ^a,b^ Means with the same letter are not significantly different (*p* < 0.05). TP total protein; CHOL, cholesterol; HDL-C, high-density lipoprotein-cholesterol; LDL-C, low-density lipoprotein-cholesterol; GLU, glucose; AST, aspartate aminotransferase; ALT, alanine aminotransferase; ALP, alkaline phosphatase; BUN, serum urea N.

**Table 7:** Effect of TSFP on the immunity of finishing pigs.

Items	3% Fish Meal	TSFP, %			SEM
		0	2.5	5	
Lymphoblasogenesis	specific fluorescence				
Lipopolysaccharide	234.25 ^b^	235.06 ^b^	262.55 ^b^	356.86 ^a^	12.67
Concanavalin A	268.88 ^b^	232.44 ^b^	241.05 ^b^	330.71 ^a^	20.23
PMA/ION	437.85	439.69	401.41	447.36	38.28
Cytokine	(pg/mL)				
Interferon-γ	115.96 ^b,c^	111.70 ^c^	122.75 ^b^	135.24 ^a^	4.39
	mean fluorescence intensity				
phagocytosis	52.04	49.36	54.32	53.75	2.81
oxygen burst	142.46 ^b^	154.46 ^a,b^	170.15 ^a,b^	180.13 ^a^	8.90
	immunoglobulin, mg/dl				
IgA	1.52 ^b^	1.59 ^a,b^	1.66 ^a,b^	1.88 ^a^	0.14
IgM	1.79	1.74	1.74	1.81	0.17
IgG	9.75	9.96	10.39	11.38	1.26
	blood T-lymphocyte, %				
CD 3	79.66 ^b^	78.59 ^b^	84.69 ^a^	85.79 ^a^	1.79
CD 4	21.86 ^a,b^	19.44 ^b^	23.74 ^a^	24.46 ^a^	1.25
CD 8	39.90	40.54	38.80	37.39	1.85
CD4^+^ CD8^+^	9.28 ^b,c^	8.85 ^c^	10.40 ^a^	10.06 ^a,b^	0.29

*n =* 20. ^a,b,c^ Means in the same row with different superscripts are significantly different (*p* < 0.05). PMA/ION: Phorbol-12-myristate-13-acetate/Ionomycin.

## Data Availability

The data presented in this study are available on request from the corresponding author.
